# Génotypes du virus de l'hépatite B et marqueurs évolutifs des patients porteurs chroniques de l'AgHBs à Bujumbura

**DOI:** 10.11604/pamj.2016.23.95.8424

**Published:** 2016-03-15

**Authors:** Rénovat Ntagirabiri, Belyse Munezero, Caritas Nahimana, Evariste Ndabaneze

**Affiliations:** 1 Centre Hospitalo-Universitaire de Kamenge, Bujumbura, Burundi; 2 Centre des maladies digestives et du Foie, Bujumbura, Burundi

**Keywords:** Virus de l′hépatite B, AgHBs, AgHBe, génotype du virus de l′hépatite B, Hepatitis B Virus, HBsAG, HBeAG, Hepatitis B virus (HBV) genotype

## Abstract

**Introduction:**

L'infection par le virus de l'hépatite B (VHB) est une affection grave suite à ses complications notamment la cirrhose et le carcinome hépatocellulaire (CHC). Les génotypes du VHB influent beaucoup sur son évolution et sur l'efficacité du traitement. Le but était d’évaluer les génotypes du VHB et les profils évolutifs des patients porteurs chroniques de l'AgHBs.

**Méthodes:**

Étude transversale, menée au Centre hospitalo-universitaire de Kamenge et au Centre des maladies du tube digestif et du foie « CEMADIF » entre Juin 2013 et Mai 2014. Le génotypage, les dosages quantitatifs de l'AgHBe et de l'ADN virale B ont été réalisés au Laboratoire Cerba, Cergy Pontoise, France. L’évaluation de la fibrose était faite par le Fibrotest ou le FibroScan.

**Résultats:**

Au total, 143 patients, 52,4% de sexe masculin, âge moyen 38,1 ans ont été inclus. Selon les marqueurs évolutifs, 112 patients (78,3%) avaient un AgHBe négatif. Quant à la charge virale, 106 patients (74,2%) avaient une virémie inférieure à 2000UI/ml et une fibrose minime inférieure à 7kpa selon le FibroScan. Parmi eux, 13 malades avaient un ADN du VHB indétectable (<20UI/ml). Les autres 37 patients (26,8%) avaient une charge virale supérieure à 2000UI/ml et parmi eux, 31 avaient un AgHBe positif (>0,8UI/ml). Il a été possible de déterminer le génotype chez 51 patients qui avaient une virémie assez élevée pour permettre techniquement ce dosage. Ces patients avaient tous un génotype A.

**Conclusion:**

Le génotype A du VHB est le plus fréquent à Bujumbura. Il est associé à un portage inactif élevé.

## Abstract (en)

**Introduction:**

Hepatitis B virus infection (VHB) is a serious condition which can lead to serious complications, such as cirrhosis and hepato-cellular carcinoma (HCC). HBV genotypes greatly influence its evolution and the effectiveness of treatment. The aim was to evaluate the HBV genotypes and the evolutionary pathways of chronic HBsAG patients.

**Methods:**

A cross-sectional study was conducted at the University Hospital of Kamenge and at the Digestive and liver diseases Center “CEMADIF” between June 2013 and Mai 2014. Genotyping, quantitative assay of HBeAG and HBV DNA levels were determined in the CERBA Laboratory Cergy Pontoise, France. Fibrotest or Fibroscan were used to evaluate fibrosis.

**Results:**

In total 33 patients (52,4% were males, median age 38,1) were enrolled. According to evolutionary markers, 112 patients (78,3%) had negative HBeAG. As regards the viral load, 106 patients (74,2%) had viremia lower than 2000UI/ml and minimal fibrosis below 7 kPa according FibroScan. Of these, 13 patients had undetectable HBV DNA (<20UI/ml). The others 37 patients (26,8%) had a viral load higher than 2000UI/ml and, among them, 31 were HBeAg positive(>0,8UI/ml). It was possible to determine genotype in 51 patients who had a high enough viremia to technically enable dosing. These patients had genotype A.

**Conclusion:**

HBV genotype-A is the most common in Bujumbura. It is associated with HBV inactive carries.

## Introduction

L'infection par le virus de l'hépatite B (VHB) constitue un problème majeur de santé publique au niveau mondial [1, 2]. Deux milliards de personnes auraient été contaminées par le VHB et 400 millions seraient des porteurs chroniques de ce virus [3]. Le VHB serait responsable de 15% à 25% de décès par ses complications notamment la cirrhose et le carcinome hépatocellulaire (CHC) [4, 5]. L'histoire naturelle de l'infection par le VHB est caractérisée par la diversité des profils clinico-virologiques allant du portage inactif au portage actif pouvant se compliquer de cirrhose ou de CHC. En effet, le VHB constitue le principal facteur de risque en Afrique [6]. Les génotypes influent sur l’évolution de la maladie et sur l'efficacité du traitement [7, 8]. Dix génotypes différents à travers le monde, désignés par des lettres de A à J et répartis selon des zones géographiques différentes ont été identifiés [8]. Dans notre pays, la prévalence du VHB est estimée à 4,6% [9]. Néanmoins à notre connaissance, les données sur les génotypes du VHB sont manquantes. En effet, aucune étude à propos des génotypes du VHB n'a été faite au Burundi. Il nous a paru pertinent de mener une première étude dont le but était d’évaluer les génotypes du VHB et les déterminants sero-immunologiques et virologiques des patients porteurs chroniques de l'AgHBs en vue d'améliorer leur prise en charge.

## Méthodes

**Type d’étude:** il s'agit d'une étude transversale, multicentrique, menée au Centre des maladies digestives et du Foie « CEMADIF » et au centre hospitalo-universitaire de Kamenge (CHUK) entre Juin 2013 et Mai 2014.

**Critères d'inclusion:** étaient éligibles à l’étude tous les patients consécutifs porteurs chroniques de l'AgHBs qui se sont présentés en consultation durant la période de l’étude. Ont été inclus les patients qui connaissaient la positivité de leur AgHBs depuis plus de 6 mois et qui ont accepté de participer dans l’étude. N'ont pas été inclus dans l’étude les patients mono-infectés par le VHB qui étaient sous traitement anti VHB; les patients co-infectés VIH-VHB qui étaient sous un schéma antirétroviral comprenant le ténofovir, la lamivudine (3TC) ou l'emtricitabine; les patients qui se connaissaient porteurs de l'AgHBs depuis moins de 6 moins ainsi que les patients qui n'ont pas souhaité participer dans l’étude.

**Déroulement de l'inclusion:** tous les patients étaient orientés au CEMADIF pour inclusion. Au CEMADIF, un médecin devait d'abord expliquer au patient le but et l'intérêt de l’étude dans l'une des trois langues (Kirundi, Swahili, Français) couramment utilisées au Burundi pour s'assurer d'une bonne compréhension par le patient. Les malades consentants étaient alors invités à signer un consentement éclairé. Après un examen clinique, ils étaient ensuite dirigés au Laboratoire du CEMADIF pour le prélèvement sanguin.

**Analyses biologiques:** Les échantillons de sang étaient conditionnés puis congelés dans les 4heures suivant le prélèvement. Certaines analyses notamment les dosages quantitatifs de l'ADN viral B (charge virale) et de l'AgHBe ainsi que la détermination du Génotype viral B ont été réalisées au Laboratoire Cerba, Cergy Pontoise, France. L’évaluation de la fibrose était faite par le Fibrotest ou le FibroScan.

**Considérations éthiques:** L’étude avait été approuvée par le Comité national d’éthique. Les analyses biologiques étaient réalisées à titre gracieux.

## Résultats

### Caractéristiques sociodémographiques des patients

Au total, 143 patients dont 52,4% de sexe masculin ont été retenus pour analyse. L’âge moyen était de 38,1 ans. Les patients provenaient de toutes les provinces du pays principalement celles de Bujumbura mairie (24,8%), Bururi (16,3%), et Bujumbura rural (13,5%). Selon les catégories professionnelles, 41,2% des patients étaient des salariés de l'Etat et 35,6% étaient des commerçants ou avaient une activité privée. La proportion des cultivateurs ou ménagères était de 8,3%, celle des élèves ou étudiants de 6,7%. Les militaires représentaient 2,7% et 5,5% étaient des chômeurs.

### Caractéristiques cliniques

Dès la connaissance de leur séropositivité à l'AgHBs, 116 patients (81,1%) nous ont déclaré avoir cessé de consommer l'alcool. Les principaux antécédents retrouvés étaient l'hypertension artérielle chez 11 patients et un diabète chez 9 malades. Selon l'index de masse corporel (IMC), 65 patients (45,5%) étaient en surpoids avec un IMC supérieur à 28kg/m^2^. A l'examen physique, 138 patients (96,5%) ne présentaient aucune anomalie clinique. Seuls 4 patients (2,8%) étaient au stade de cirrhose dont un avait avec un CHC.

### Déterminants séro-immunologique et virologique

Les transaminases ont été dosées une seule fois à l'inclusion. Elles étaient normales chez 112 patients (74,2%). Selon les marqueurs sérologiques, tous les patients avaient un AgHBs positif, des Ac Anti-HBc positifs et des Ac anti-HBs négatifs. 112 patients (78,3%) avaient un AgHBe négatif et des AC anti-HBe positifs. Les 31 autres patients (21,7%) avaient un AgHBe positif avec un pic quantitatif de 723,7 UI/ml. Selon la charge virale, 106 patients (74,2%) avaient une virémie inférieure à 2000UI/ml et une fibrose minime, inférieure à 7 kpa selon le FibroScan. Parmi eux, 13 malades (12,3%) avaient un ADN du VHB indétectable (<20UI/ml). A l'inverse, 37 patients avaient une charge virale supérieure à 2000UI/ml et la plus élevée était à 41.968.311UI/ml. Parmi ces 37 patients, 31 soit (83,7%) avaient un AgHBe positif (>0,8UI/ml). Les profils évolutifs des patients sont résumés dans le Tableau 1.

**Tableau 1:** Génotypes du VHB et déterminants immuno-virologiques des patients

Profil évolutif	Variables analysées					Effectif
	AgHBs	AgHBe	ADN/VHB	Transaminases	Génotype VHB	
Portage inactif	positif	<0,8UI/ml	<2000 UI/ml	Normales	A (n* = 14)	106
V sauvage actif	positif	>0,8UI/ml	>2000 UI/ml	Elevées	A (n* = 31)	31
V mutant actif	positif	<0,8UI/ml	>2000 UI/ml	Elevées	A (n* = 6)	6

ADN/VHB: ADN quantitatif du virus de l'hépatite B; V: virus

* n: nombre de patients chez qui la détermination du génotype a été possible

**Génotype du VHB:** Il a été possible de déterminer le génotype du VHB chez 51 patients qui avaient une virémie permettant techniquement ce dosage. Ils avaient tous un génotype A.

## Discussion

Dans notre étude, la détermination du génotype a été possible chez 51 malades. Les autres patients n'avaient pas une virémie suffisamment élevée pour pouvoir le faire. Ainsi, cette première étude a montré que le génotype A est le plus fréquemment rencontré dans cette population de 51 malades provenant des 5 régions du pays à des proportions différentes. En effet, selon la littérature, le génotype A est le plus fréquent en Afrique sub-saharienne [8]. Il aurait une évolution favorable sous traitement anti-viral et serait sensible à l'interféron [8]. Cette étude a également montré que le profil de porteur inactif du VHB est le plus fréquent. En effet, plusieurs auteurs ont rapporté que les profils évolutifs porteurs chroniques du VHB ainsi que les mutations virales et les séroconversions anti-HBe dépendaient largement des génotypes [8, 10]. Ainsi Lindh et al ont trouvé que le portage chronique du génotype C était plus associé à un taux élevé de l'AgHBe positif, à une virémie importante, à l'existence d'une activité inflammatoire du foie et à une fibrose sévère que le portage du génotypes A ou des autres génotypes [10]. Cela pourrait expliquer en partie la proportion élevée des patients ayant un profil de porteurs inactifs dans notre étude. En outre, Livingston et al, dans une série de 1158 patients suivis sur une longue période, ont trouvé que les porteurs du génotype A avaient plus de séroconversion anti-HBe par rapport aux porteurs du génotype C [11]. A l'inverse, les patients porteurs du Génotype C après une séroconversion anti-HBe, avaient une grande incidence de réactivation de l'AgBHe [11]. Une autre donnée intéressante décrite dans la littérature est que la médiane de clearance de l'AgHBe chez les porteurs chroniques du génotype A est de 20 ans alors qu'elle est de 48ans pour le génotype C [11]. Cela a des implications et pourrait laisser penser que dans notre pays où semble prédominer le génotype A, les femmes gestantes porteuses chroniques de l'AgHBs transmettraient moins le VHB en périnatale. En effet, la contamination se faisant au bas âge en Afrique en général et par conséquent au Burundi, ces gestantes auront fait une séroconversion anti-HBe et la contagiosité aura baissé à l’âge adulte. Cette séroconversion précoce liée au génotype A est retrouvée dans notre étude car en effet 84,3% de nos patients avaient un AgHBe négatif avec un âge moyen de 38,1ans. Toutefois, le taux de séroconversion liée au génotype A serait inférieure à celle du génotype D [12]. Quant à la réplication virale, des études notamment celle menée en 2006 chez les enfants, ont montré que le portage du génotype A était également liée à une faible virémie [10, 12]. Ces données de la littérature corroborent nos résultats. En effet, 74,1% des patients de notre série avaient une charge virale inférieure à 2000UI/ml et parmi eux 12,3% avaient une charge virale indétectable (<20UI/ml). Il est connu qu'une virémie élevée est un des facteurs de risque potentialisant une évolution vers la cirrhose et le CHC [13]. Bien que le génotype A soit associé à un bon profil évolutif, il ne constitue pas un facteur suffisant de bonne évolution. Il existe des facteurs notamment ceux liés à l'hôte influençant l’évolution vers les complications [13]. En effet, une étude récente de suivi prospective d'une cohorte de patients porteurs chroniques de l'AgHBs sur une période de 5 ans, a montré une incidence annuelle de CHC estimée à 1,8% dans notre pays [14]. La présence de cirrhose multipliait par 10 le risque de survenue de CHC et l'incidence annuelle s’élevait à 7,1% [14].

## Conclusion

Le génotype A du VHB est le plus fréquent dans notre population d’étude. Il est associé à un taux de portage chronique inactif élevé. Cependant, il convient de bien surveiller les patients porteurs chroniques de l'AgHBs même si ce génotype prédominant semble être le moins agressif.

### Etat des connaissances sur le sujet

- Il est connu que le virus de l'hépatite B constitue un problème majeur de santé publique.
- En outre, il existe plus de dix génotypes dans le monde qui influent sur l’évolution de la maladie et sur l'efficacité du traitement.
- Néanmoins, il n'y a pas de données au Burundi à propos des génotypes du virus de l'hépatite B et du profil évolutif des patients selon le génotype porté.

### Contribution de notre étude a la connaissance

- L’étude apporte des connaissances nouvelles qui montrent que le génotype A du virus de l'hépatite B est le plus fréquent dans notre population d’étude.
- Il est associé à un taux de portage chronique inactif élevé, estimé à 74,2%.
